# High-sensitivity plasma proteomics reveals disease-specific signatures and predictive biomarkers of Alzheimer’s disease phenotypes in a large mixed dementia cohort

**DOI:** 10.21203/rs.3.rs-6440485/v1

**Published:** 2025-06-29

**Authors:** Katherine Gong, Jigyasha Timsina, Muhammad Ali, Yike Chen, Menghan Liu, Ciyang Wang, Cyril Pottier, Geoffrey K. Feld, Gyujin Heo, Tammie L. S. Benzinger, Cyrus A. Raji, Beau Ances, Brian A. Gordon, Julie K. Wisch, Suzanne E. Schindler, John C. Morris, David M. Holtzman, Laura Ibanez, Carlos Cruchaga

**Affiliations:** Washington University School of Medicine; Washington University School of Medicine; Washington University School of Medicine; Washington University School of Medicine; Washington University School of Medicine; Washington University School of Medicine; Washington University School of Medicine; Geocyte LLC; Washington University School of Medicine; Washington University School of Medicine; Washington University School of Medicine; Washington University; Washington University; Washington University; Washington University School of Medicine; Washington University School of Medicine; Washington University School of Medicine; Washington University School of Medicine; Washington University School of Medicine

**Keywords:** Plasma assays, Neurodegeneration diagnostics, Plasma-based diagnostics, CNS protein profiling

## Abstract

Highly sensitive plasma assays enable accurate blood-based biomarkers for neurodegenerative disease and provide minimally invasive options for clinical use. Large-scale studies encompassing multiple neurodegenerative diseases and utilizing multiplex platforms are essential to uncover disease-specific biomarkers and pathways.

We generated and analyzed plasma proteomics using the NULISASeq^™^ CNS Disease Panel 120 from 3,002 participants with Alzheimer disease (AD), Dementia with Lewy bodies (DLB), Frontotemporal dementia (FTD), Parkinson disease (PD) and cognitively unimpaired participants at the Charles F. and Joanne Knight Alzheimer Disease Research Center. We identified proteins associated with disease status and AD-related phenotypes (Clinical Dementia Rating^®^ [CDR^®^], CSF Aβ42/Aβ40, amyloid-PET, and tau-PET tauopathy), which were used to identify disease-specific biomarkers and perform pathway analyses.

Among the 123 measured protein, 78 were associated with AD, two with DLB, two with FTD, and one with PD after multiple test correction. Disease comparison showed that AD and DLB showed the highest similarity, followed by FTD and DLB. At the same time there were also disease-specific signatures. Some AD-specific proteins include p-tau217 being AD-specific, MME was specific for FTD, CHR for DLB and PARK7 for PD. We also identified 8 proteins associated with Amyloid PET, 7 with Tau PET, 14 with CSF Aβ42/40 ration and 73 with CDR, with Amyloid PET and CDR showing the highest overlap. As few extensive plasma p-tau217 studies have been performed with the NULISA platform, we used a data-driven approach to establish the cut-off for biomarker positivity, and analyze its predictivity performance for clinical status and amyloid-PET. Plasma p-tau217 achieved an AUC of 0.81 (95% CI: 0.79−0.83) for AD diagnosis and 0.95 (95% CI: 0.93−0.98) for amyloid positivity. Using a two-cutoff approach, plasma p-tau217 had an AUC of 0.95 and 93.59% agreement with amyloid-PET status. Proteins associated with AD were enriched on vascular endothelial growth factor receptor binding, mainly driven by VEGFD and VEGFA. Cell death and apoptosis pathways were unique to FTD and driven by CCL2 and TREM2, and PD was enriched on enzymatic activity and metal ion binding.

This is the largest plasma proteomic investigation to date, incorporating p-tau217 and utilizing the NULISA platform to understand neurodegenerative diseases. It validates the high classification accuracy of plasma p-tau217 and its strong correlation with amyloid PET status. Additionally, we identify disease-specific proteins that could enhance differential diagnosis. These findings underscore the potential of the NULISA platform as a reliable quantitative tool for research and clinical applications in neurodegenerative diseases.

## Introduction

Neurodegenerative diseases such as Alzheimer disease (AD), Parkinson disease (PD), frontotemporal dementia (FTD), and dementia with Lewy bodies (DLB) affect millions of people worldwide. With the increase in global life expectancy, the social and economic burden of neurological disorders is rising.^1–3^ Although many types of treatment targets have been evaluated,^4^ the only disease-modifying treatments thus far that have definitively demonstrated efficacy in slowing cognitive decline are anti-amyloid antibodies that target amyloid pathology.^5^ To enable development of additional types of treatments, novel biomarkers are needed to identify individuals with different pathologies. Biomarkers requiring Positron Emission Tomography (PET) scans or cerebrospinal fluid (CSF) are burdensome to patients and providers and require specialized personnel and/or equipment. Blood-based biomarkers are more practically useful because of their minimal invasiveness and the potential for integration into routine clinical practice.^6,7^

Numerous studies have explored the use of plasma biomarkers in neurodegenerative diseases.^8–13^ Plasma p-tau217 has demonstrated high accuracy in detecting AD pathophysiology within the context of routine clinical practice in a memory clinic.^8^ In addition, plasma biomarkers such as the ratio of amyloid beta (Aβ)42 to Aβ40, neurofilament light (NEFL/NfL), and glial fibrillary acidic protein (GFAP) hold significant potential for AD diagnosis and monitoring.^9,10^ Similarly, plasma proteomic analysis in PD has revealed multiple novel biomarkers, such as DOPA decarboxylase (DDC). However, plasma DDC concentrations may be impacted by some medications^11^. The heterogeneity and low prevalence of FTD has hindered the identification of clinically useful biomarkers. Nevertheless, recent research suggests that plasma extracellular vesicles (EVs) content may serve as potential diagnostic biomarkers.^12^ Chatterjee *et al.* demonstrated that EVs in plasma contain quantifiable amounts of TAR DNA-binding protein (TDP-43) and tau isoforms, with distinct patterns observed in diseases like Amyotrophic lateral sclerosis (ALS) and FTD. Their findings show that EV TDP-43 levels and 3R/4R tau isoform ratios effectively discriminate between diagnostic groups and strongly correlate with disease severity, highlighting their potential as biomarkers for FTD disease monitoring.^12^ DLB is a common form of cognitive neurodegenerative disease, yet only one-third of patients receive accurate diagnoses, primarily due to its clinical similarities with AD and PD. Several studies have proposed p-tau181 as a potential plasma biomarker for DLB, while AD non-specific biomarkers such as NEFL and GFAP also show increased levels across the disease continuum.^13^

However, most biomarker studies focused on a single disease and examined a limited set of plasma protein markers. There is a pressing need for large-scale studies that compare multiple plasma biomarkers across AD, PD, DLB, and FTD, while also identifying novel disease-specific biomarkers. The newly developed NUcleic acid Linked Immuno-Sandwich Assay (NULISA) platform balances between multiplexing, low volume, and accuracy. It has limited cross-reactivity issues seen in other multiplexed immunoassay platforms.^14^ We have previously demonstrated the reliability of the NULISA measurements for biomarker measurement in AD ins a small datasets (n=48), showing strong correlations with established assays in both CSF and plasma.^15,16^ Leveraging the multiplex capacity of this platform, we aim to explore proteomic abundance patterns across various neurodegenerative diseases to identify novel dysregulated proteins and assess their potential as biomarkers. In this study, we utilized the NULISA CNS Disease Panel 120, which includes validated AD biomarkers (p-tau217, p-tau181, TREM2,) alongside key proteins associated with PD (SNCA, pSNCA-129), FTD (TDP-43), and general neurodegeneration (NEFL, GFAP, NRGN, SMOC1). Our large, well-characterized cohort allowed us to evaluate the predictive power of these proteins across diseases and identify novel disease-specific biomarkers. Through pathway analysis, we sought to uncover the biological mechanisms driving disease progression. Finally, we assessed the platformś reproducibility and cross-validated its performance against other orthogonal platforms.

## Materials and methods

### Ethics statement

The study was approved by the Institutional Review Board of Washington University School of Medicine in St. Louis (IRB #201109148) and conducted in accordance with approved protocols. Ethics approval for individual cohorts was obtained from their respective IRBs, with written informed consent provided by participants or their families

### Study design

In this study, we investigated alterations in the plasma proteome measured using NULISA to identify proteomic signatures associated with different neurodegenerative diseases (AD, FTD, DLB and PD). We measured 123 unique proteins included in the NULISAseq CNS Disease Panel 120. A total of 3,746 human plasma samples were collected, and after quality control (QC), 3,668 samples from 3,002 unique participants remained. These participants were enrolled at the Charles F. and Joanne Knight Alzheimer Disease Research Center (Knight-ADRC). To identify disease-specific proteomic changes, differential abundance analysis was performed comparing each disease group to healthy controls. Significant features were then used as input to perform pathway analysis. Similar analyses were performed for four AD-related phenotypes: amyloid-PET (*N* = 280), Tau PET (*N* = 264), CSF Aβ42/Aβ40 (*N* = 504), and Clinical Dementia Rating (CDR; *N* = 2,671). Finally, we evaluated the reproducibility of results between runs using 86 identical samples and the correlation of protein measurement with other proteomic measurement technologies like immunoassay (*N* =1,414) and SomaScan (*N* = 3,622).

### Cohort

#### Sample collection

Plasma samples were immediately processed after collection and stored at −80°C until use. Prior to measurement, samples were thawed and centrifuged at 10,000g for 10 min. Then, 10 μL supernatant from each sample was plated in 96-well plates and assayed with the NULISAseq CNS Disease Panel 120 v1.

#### Proteomics data measurement and quality control

Protein expression levels were measured using the NULISAseq CNS panel, which targets 123 proteins, as previously described.^24^ Protein concentrations, reported in NULISA Protein Quantification (NPQ) units, are normalized for intraplate and intensity variability, then log2-transformed to approximate a normal distribution. No further transformations were applied.

As part of the quality control (QC) procedure, data points falling outside 1.5 times the interquartile range (IQR) from the first (Q1) or third (Q3) quartile were flagged as outliers and replaced with NAs. Subsequently, call rates (the proportion of successful measurements) were calculated for each analyte and sample. A two-step call rate threshold filter was applied, starting with 65%, followed by 85%, to identify and remove relatively low-quality data. The call rate was recalculated after the 65% (high rate of missingness) filter and before applying the 85% filter. This two-step approach was designed to retain borderline analytes and samples. The final dataset consisted of 123 analytes and 3,668 samples (3,002 unique participants) after QC. Limit of detection (LOD) and coefficient of variation (CV) were assessed for each analyte; however, these parameters were not used as filters. Following QC, the default log2-based NPQ values were back-transformed to linear NPQ to allow end users to apply their preferred normalization. For this study, linear NPQ values were log10 transformed.

#### Technical comparison

To assess the technical robustness and reproducibility of the NULISAseq CNS Disease Panel 120, measurements were first evaluated across multiple runs (*N* = 86) and under varying matrix conditions (*N* = 43; paired samples collected in Ethylenediaminetetraacetic acid (EDTA) and sodium citrate). Further comparisons were performed by correlating NULISAseq measurements with those from SomaScan (*N* = 3,622) and immunoassay (*N* = 1,414). Proteins included in the analysis were based on data availability or presence in both platforms being compared, with common proteins identified based on their UniProt ID.

#### Neuroimaging Methods

Magnetic resonance images (MRI) were obtained on 3T Siemens scanners. T1-weighted scans were segmented using FreeSurfer 5.3 (Martinos Center for Biomedical Imaging, Charlestown, Massachusetts, USA) using the Desikan-Killiany atlas. Positron emission tomography (PET) scanning for amyloid was performed using either [11C] Pittsburgh Compound B (PiB) and [18F] Florbetapir, following previously described methods^17^, and was processed using the MR-Free pipeline.^18^ Individuals were classified as amyloid-positive if they had a cortical amyloid burden above 20 Centiloids (CL).^19^ PET scanning for tau was performed using [18F] Flortaucipir following previously described methods.^20^ Images were processed using the publicly available PET unified pipeline (PUP, https://github.com/ysu001/PUP).^17,21^ Individuals were considered tau positive if they had a partial-volume corrected SUVR greater than 1.5 in the tau signature region (arithmetic mean of tau SUVR in the amygdala, entorhinal cortex, inferior parietal region, and lateral occipital cortex).^22^ Cerebellar grey matter served as the reference region for all PET image processing.

#### Cerebrospinal Fluid Collection

CSF samples were collected at approximately 8:00 AM following overnight fasting.^23,24^ CSF Aβ40 and Aβ42 concentrations were measured by chemiluminescent enzyme immunoassay using a fully automated platform (LUMIPULSE G1200, Fujirebio, Malvern, PA, USA). Individuals were considered amyloid positive if their CSF Aβ42/Aβ40 ratio was less than 0.0673.^23^

### Statistical Analysis

#### Differential abundance analysis

Proteins associated with each disease were identified using linear regression analysis, with clinical cases (AD, DLB, FTD, and PD) compared to clinical controls, which served as the reference group in all comparisons. The regression model was adjusted for age at plasma collection, sex, and the first two surrogate variables (SVs) as covariates to account for potential confounding factors.

SVs were calculated using the "sva"function from the "sva (v 3.52.0)" package in R, with missing expression values imputed via random sampling^25^. A linear regression model was implemented for four AD-related phenotypes: amyloid-PET, Tau PET, CSF Aβ42/Aβ40, and CDR. FDR-adjusted p-value < 0.05 was a defined significance for these comparisons. The differential abundance analysis results were visualized using volcano plots generated using "ggplot (v3.5.1)"^26^. Pearsońs correlation was used to compare protein effect sizes across analyses within the disease and phenotype comparison separately.

#### Plasma p-tau217 cutoff determination

To establish robust p-tau217 cutoffs for classifying biomarker positivity, we applied a Gaussian Mixture Model (GMM) clustering using the "mclust (v6.0.0)" package^27,28^ in R, a method we have previously shown to be effective for identifying data-driven cutoffs.^29^ Briefly, we first calculated z-scores (mean = 0, variance = 1) from the log10-transformed p-tau217 values. GMM was then used to identify two normal distributions within the dataset. The cutoff was set at the z-score (and its corresponding raw value) where a sample was equally likely to belong to either distribution. We also determined a single cutoff using Youdeńs Index to balance sensitivity and specificity.

Previous studies suggested that a two-cutoff approach with an intermediate group improves classification^30,31^. Using p-tau217 and amyloid-PET data, we derived upper and lower cutoffs corresponding to 95% sensitivity and specificity, respectively. An amyloid-PET value of 20 was used to categorize samples as positive (>20) or negative (<20), as previously reported for Knight ADRC samples.^19^ Similarly, we applied this strategy to determine the p-tau217 cutoff for Tau PET, using a threshold of 1.5^22^ to define Tau PET levels. The concordance of these p-tau217 cutoffs with amyloid-PET and Tau PET statuses were then evaluated to ensure robustness.

#### Progression to symptomatic AD

We performed a survival analysis using Cox proportional hazards regression model to identify proteins that are associated with progression of individuals to symptomatic AD. The analysis was adjusted for age at plasma draw and sex and was implemented through the "survival (v3.5.5)" R package. Individuals who were cognitive intact at the time of sample collection but progressed to symptomatic AD cases during follow-up were compared against those who remained unimpaired; the latter group served as the reference. Time-to-event was calculated from the difference between the age at blood draw and the age at last follow-up (for controls) or the age of AD onset (for cases). Significance was defined by an FDR-adjusted p-value < 0.05. Samples were divided into high and low groups based on the GMM p-tau217 cutoff described in an earlier section, and survival time were compared using the "adjustedCurves" R package.^32,33^

#### Predictive models

To assess the ability of individual proteins to distinguish cases from controls, the receiver operating characteristic (ROC) curve and area under the curve (AUC) values were calculated as described elsewhere^34^. Briefly, multivariate logistic regression, adjusted for age at plasma draw and sex, was performed with binary clinical status (cases vs controls) as the response variable and protein values as the predictor variable. In the model evaluating the predictive power of p-tau217, the response variable was based on whether individuals progressed to disease within different time frames.

#### Pathway Analysis

Pathway enrichment analysis was conducted using the "clusterProfiler (v4.12.0)" package in R^35,36^. The "enrichGO" function within the package was used to identify overrepresented pathways based on Gene Ontology (GO) annotations. For analyses with a single or very few significant features, we relaxed the significance threshold to p < 0.05, allowing more features to be included as input. The Entrez gene IDs corresponding to genes matching the protein list were used as input. Significant pathways were defined as those that passed FDR-adjusted p < 0.05.

#### Correlation between runs and with other platforms

The reproducibility of measurements and comparison of the NULISA platformś performance to other proteomic platforms were assessed using Pearsońs correlation coefficient. Significant correlations were defined as those with p < 0.05.

## Results

### Study Participants

This study included 3,668 plasma samples collected from 3,002 unique participants recruited at the Charles F. and Joanne Knight Alzheimer Disease Research Center (Knight ADRC) at Washington University in St. Louis. These 3,002 participants included 1,092 AD cases, 39 FTD cases, 28 DLB cases, 9 PD cases, and 1,579 cognitively unimpaired controls (CO) based on the diagnosis on the last visit (Table 1). The mean age (±standard deviation; *SD*) at plasma draw was 77.68 ± 8.37 for AD, 75.46 ± 11.57 for DLB, 77.11 ± 6.79 for PD, 67.28 ± 9.45 for FTD, and 72.88 ± 10.58 for CO. The proportion of males varied across groups, with the highest percentage observed in the PD group (88.89%), followed by DLB (64.29%), FTD (56.41%), AD (43.68%), and CO (40.22%). The mean age of disease onset (±SD) was 73.20 ± 8.84, 70.71 ± 11.64, 72.67 ± 9.64, and 63.10 ± 9.88 for AD, DLB, PD, and FTD, respectively. The prevalence of *APOE4*+ participants was highest in the AD group (58.33%), followed by FTD (46.15%), DLB (42.86%), PD (33.33%), and CO (30.72%; Table 1). Additional AD-related phenotypes included amyloid PET (*N* = 280), Tau PET (*N* = 264), CSF Aβ42/Aβ40 (*N* = 504), and Clinical Dementia Rating^®^ (CDR^®^; *N* = 2,671, Table 2).

### Proteomic Signatures in Plasma across neurodegenerative disease

**Figure 1:**
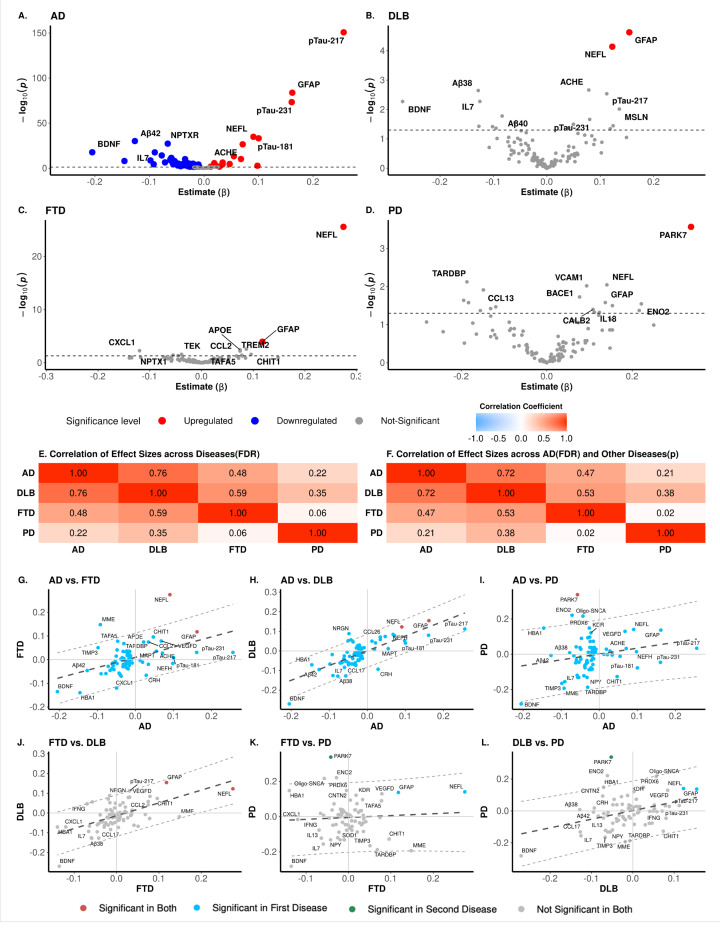
Plasma proteins associated with AD and ADRD. **(A-D)** The volcano plots illustrate proteins with significantly different abundances between cognitively healthy controls (CO) and patients with Alzheimer’s disease (AD), dementia with Lewy bodies (DLB), frontotemporal dementia (FTD), or Parkinsońs disease (PD). Each point corresponds to a protein, with colored points denoting those with significant abundance differences, identified by an adjusted p-value of < 0.05. The x-axis represents the effect size, while the y-axis depicts the -log10 of the raw p-value, highlighting proteins with both substantial and statistically significant abundance changes. **(E)** The heatmap illustrates the correlation of effect sizes for proteins significantly associated (FDR < 0.05, n = 78) across various disease types. **(F)** The heatmap displays the correlation of effect sizes for proteins significantly associated with AD (FDR < 0.05) and other diseases (P < 0.05, n = 93) across various disease types. **(G-L)** Scatter plots comparing effect sizes of significant proteins from either dataset. Proteins are selected if they are significant in at least one analysis. Red points indicate significance in both datasets, blue points indicate significance in the first disease, green points indicate significance in the second disease, and grey points indicate no significance in either dataset. The dark dashed grey line represents the regression line, while the light grey dashed line outlines the confidence interval ribbon, indicating the confidence bounds of the prediction.

We performed linear regression analyses to identify proteins associated with AD (*N* = 1,092), DLB (*N* = 28), FTD (*N* = 39), and PD (*N* = 9; Table 1). Among the 123 protein analytes measured, 78 were found to be associated with AD, two with DLB, two with FTD, and one with PD after FDR correction (Fig. 1A–D, Supplementary Table 1–4). Two analytes, GFAP and NEFL, were associated with AD, DLB, and FTD, while PARK7 was found to be associated with AD and PD (Fig. 1A–D, Supplementary Fig. 1). P-tau217 was the most significantly associated protein with clinical AD status (*β*= 0.26; *p* = 1.99×10^−149^). In addition, it was nominally significant for DLB, but not for PD or FTD. Other highly significant proteins for AD included GFAP (*β*_*AD*_= 0.16; *P*_*AD*_= 9.63×10^−83^), p-tau231 (*β*_*AD*_= 0.16; *p*_*AD*_= 2.02×10^−72^), NEFL (*β*_*AD*_= 0.09; *p*_*AD*_= 4.02×10^−34^) and p-tau181 (*P*_*AD*_= 0.09; *p*_*AD*_= 1.59×10^−32^; Supplementary Table 1–4). Our analysis also captured AD-associated proteins known to be involved in AD-related pathways, including full-length MAPT, APOE, PSEN1, and BACE, as well as proteins reported to be associated with AD in some large-scale proteomics studies, include NPTXR, ACHE, BDNF, IL7, VEGF-D and VEGF-A^37–39^. Interestingly, proteins known to be implicated in neurodegenerative diseases other than AD, such as SNCA, pSNCA-129, Oligo-SNCA, SOD1, and TARDBP were also significantly associated with AD. In addition, some proteins reported to be associated with AD in CSF^39^ did not show associations in plasma, including TREM2, YWHAZ, NPTX1, SMOC1, YKL40.

Only NEFL (*β*_*FTD*_ = 0.27; *P*_*FTD*_ = 3.29×10^−24^; *β*_*DLB*_= 0.12; *P*_*DLB*_= 4.46×10^−3^) and GFAP (*β*_*DLB*_= 0.15; *p*_*DLB*_= 2.89×10^−3^; *β*_*FTD*_= 0.12; *p*_*FTD*_= 6.75×10^−3^) were associated with DLB and FTD after multiple test correction. Both were also significantly associated with AD. Other nominally assocaited proteins included TREM2, APOE, CXCL1, and CCL2 for FTD, as well as ACHE, Aβ38, p-tau217, and IL7 for DLB. (Supplementary Table 2, 3). In PD, the only FDR-adjusted significantly differential protein was PARK7 (*β* = 0.34; *p* = 3.31×10^−2^), with TARDBP, NEFL, and VCAM1 showing nominal associations (Supplementary Table 4).

To further evaluate how similar or different AD, FTD, DLB, and PD are, we assessed the correlations of effect sizes across these diseases (Fig. 1E–L, Supplementary Fig. 2). The strongest correlation was observed between AD and DLB (*r* = 0.76, *p* = 6.88×10^−16^), indicating a significant overlap in the proteomic profile underlying these two neurodegenerative diseases. To determine what proteins were leading to these results, we evaluated which proteins had consistent effect size and which did not by analyzing those proteins that were outside of the 95% CI for the effect size correlation interval (Fig. 1G–L). In this analysis, the 95% CI was used as a baseline to identify proteins with effect sizes that deviated significantly from the overall distribution. We have previously used a similar approach to compare the proteomic profiles between autosomal-dominant and sporadic AD.^38^ When comparing AD and DLB, all but five proteins were within the 95% CI. Among the proteins outside this boundary, BDNF showed a larger absolute effect size in DLB than in AD. Notably, four proteins exhibited opposite effect sizes between AD and DLB. CRH showed a positive effect size in AD but negative in DLB, while NRGN, CRP, and Oligo-SNCA showed negative direction of change in AD but positive in DLB.(Supplementary Table 1, 2) A moderate correlation was observed between AD and FTD (*r* = 0.48, *p* = 7.30×10^−6^). In this comparison, NEFL showed higher effect size in FTD than in AD, and MME showed discordant effect sizes. Finally, the correlation between DLB and PD was notably the weakest (*r* = 0.35, *p* = 1.51×10^−3^), as this difference was driven by PARK7, which was also different when comparing PD with FTD and AD. PARK7, together with Oligo-SNCA and ENO2 also showed opposite effect size between AD and PD. These data suggest that increased levels of PARK7 are specific to PD, while it is also significant in AD, but in the opposite direction. Oligo-SNCA is capturing synuclein pathology as is associated with PD and DLB; NEFL is a better predictor for FTD, and p-tau217 for AD. As the statistical power for DLB, FTD and PD is limited, we also extended these results to include any protein that showed nominal association with these diseases, and these analyses supported the previous cross-disease correlations (Fig. 1F).

**Figure 2:**
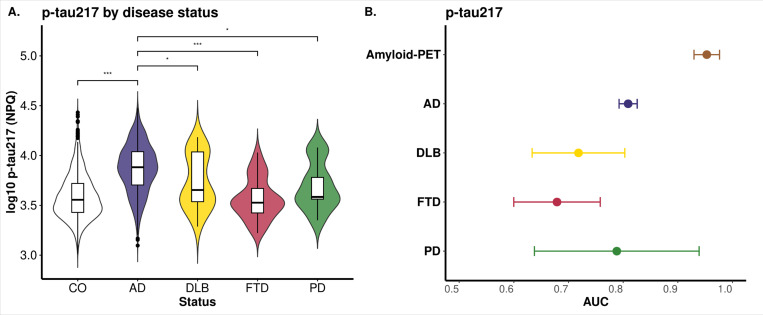
Plasma p-tau217 Expression Across Diseases. **(A)**The violin plot illustrates the distribution of log10 p-tau217 (NPQ values) across various classified statuses, including cognitively healthy controls (CO), Alzheimer‘s disease (AD), and other conditions, with significance annotations highlighting statistically significant differences in p-tau217 levels between these conditions. **(B)** The graph assesses the predictive power of p-tau217, Sex, and Age for various conditions using logistic regression. It displays the AUC (Area Under the Curve) and confidence intervals for each dataset, highlighting the effectiveness of these predictors.

We also performed additional sensitivity and specificity analyses across AD and Alzheimer’s Disease and Related Dementias (ADRDs) for p-tau217 since it was the most significantly associated protein with clinical AD status. Plasma p-tau217 levels showed significant differences between AD and CO (*p* < 2×10^−16^), as well as between AD and FTD (*p* = 6.20×10^−15^), AD and DLB (*p* = 5.90×10^−3^), and AD and PD (*p* = 3.46×10^−2^; Fig. 2A). A detailed analysis of the p-tau217 distribution in FTD, DLB, and PD reveals a bimodal pattern, with some individuals exhibiting p-tau217 levels similar to those in AD. This observation suggests that there is a percentage of DLB, FTD and PD samples with amyloid pathology or a potential misdiagnosis. When we evaluated the predictive power of p-tau217 across these diseases, the AUC for clinical AD was 0.81, which was significantly higher than that for DLB (*AUC* = 0.72) and FTD (*AUC* = 0.68), suggesting that in fact, p-tau217 might be an AD-specific marker (Fig. 2B).

### AD endophenotypes show differences in proteomic signatures

The large, well-characterized AD cohort that includes longitudinal data facilitated the identification of potential protein biomarkers predictive of AD-related phenotypes: amyloid-PET (*N* = 280), tau PET imaging (*N* = 264), CDR (*N* = 2,671) and CSF Aβ42/Aβ40 ratio (*N* = 504, Table 2).

**Figure 3:**
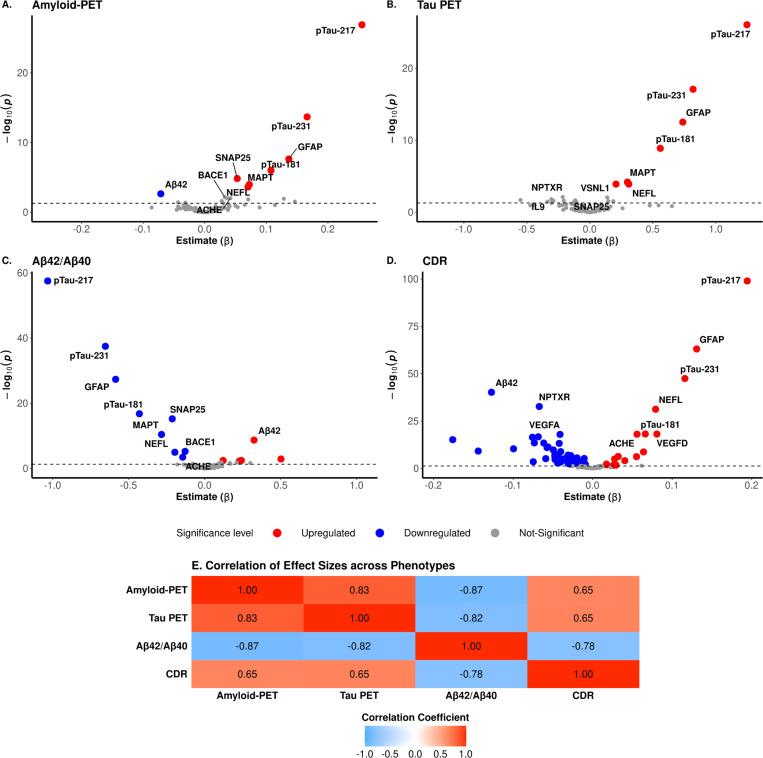
Association of plasma proteins with AD endophenotypes. **(A-D)** The volcano plots illustrate proteins with significantly different abundances in Alzheimer’s disease (AD) and control (CO) groups, stratified by amyloid-PET, Tau PET, CSF Aβ42/Aβ40, and CDR levels. Each point corresponds to a protein, with colored points denoting those with significant abundance differences, identified by an adjusted p-value of < 0.05. The x-axis represents the effect size, while the y-axis depicts the -log10 of the raw p-value, highlighting proteins with both substantial and statistically significant abundance changes. **(E)** The corresponding heatmap presents the correlation of effect sizes for these significant proteins across multiple phenotypic measures.

We identified eight proteins associated with amyloid-PET levels, seven with Tau PET imaging, 14 proteins with the CSF Aβ42/Aβ40 ratio, and 73 proteins with CDR (Fig. 3A–D, Supplementary Table 5–8), with p-tau217 being the largest contributor for all four phenotypes. Additional AD-related markers, including p-tau231, p-tau181, MAPT, GFAP, and NEFL, were also significant across all phenotypes. Like the comparison across the diseases, we assessed the uniqueness of proteomic signatures for each AD phenotype using effect size and protein level correlations. (Fig. 3E, Supplementary Fig. 3A-F). The strongest absolute correlation was observed between Aβ42/Aβ40 and amyloid-PET (*r* = −0.87, *p* = 1.70×10^−24^; Fig. 3E), suggesting that CSF Aβ42/Aβ40 and amyloid PET are capturing similar proteomic signatures. All but two proteins showed similar effect sizes across these two phenotypes. HBA1 showed a higher effect size for amyloid PET than CSF Aβ42/Aβ40, suggesting that this could be an amyloid-PET- or PET-specific marker. We also found strong correlations in effect size between Tau-PET and amyloid PET (*r* = 0.83) and CSF Aβ42/Aβ40 (*r* = −0.82). Again, HBA1 also showed larger effect size for Tau-PET than for CSF Aβ42/Aβ40, whereas on the other hand, MME seemed to be an amyloid-specific protein as it showed higher effect size for both CSF Aβ42/Aβ40 and amyloid PET. The weakest correlation was observed between CDR and amyloid-PET (*r* = 0.65) and tau-PET (*r* = 0.65; Fig. 3E), and the proteins that are leading to lower correlation seem to be shared and driven by HBA1 and ARSA. BDNF and TIMP3 showed lower effect size for amyloid-PET compared to CDR but not for Tau-PET. On the other hand, VEGFD showed a significantly higher effect size in CDR compared to Tau-PET but not compared to amyloid-PET.

Since, pTau217 was the most significant association in all four phenotypes, we assessed its predictive power for these phenotypes. ROC analysis showed p-tau217 achieved an AUC of 0.81 for AD status and 0.95 for amyloid-PET (Fig. 2B). Then, we analyzed if combining p-tau217 with protein ratios from Aβ42, BDNF, and NPTXR, which showed the largest effect sizes in the opposite direction, could enhance predictive power. The p-tau217/NPTXR ratio showed the best performance (*AUC* = 0.86; 95% *CI:* 0.84−0.87) for clinical AD status, followed by the p-tau217/Aβ42 ratio (*AUC* = 0.84, 95% *CI:* 0.83−0.86), both besting the standalone p-tau217 model (*AUC* = 0.81; Supplementary Fig. 4). The p-tau217/BDNF ratio showed the poorest performance AUC (0.74, 95% *CI*: 0.73−0.76). For amyloid-PET classification, standalone p-tau217 achieved the highest AUC (0.94), closely followed by p-tau217/Aβ42 and p-tau217/NPTXR (*AUC* = 0.94 and 0.93; respectively; Supplementary Fig. 4). Overall, we observed that p-tau217 showed strong predictive ability in distinguishing between clinical AD and CO, as well as in classifying amyloid-PET biomarker positive and negative participants. However, combining p-tau217 with other markers enabled a more comprehensive prediction of clinical disease.

### Plasma p-tau217 as a biomarker of brain amyloidosis

Given the strong association between p-tau217 and amyloid-PET, we evaluated the predictive power of plasma p-tau217 for amyloid-PET positivity and used multiple approaches to establish potential cutoffs for plasma p-tau217 levels measured using NULISAseq.

**Figure 4:**
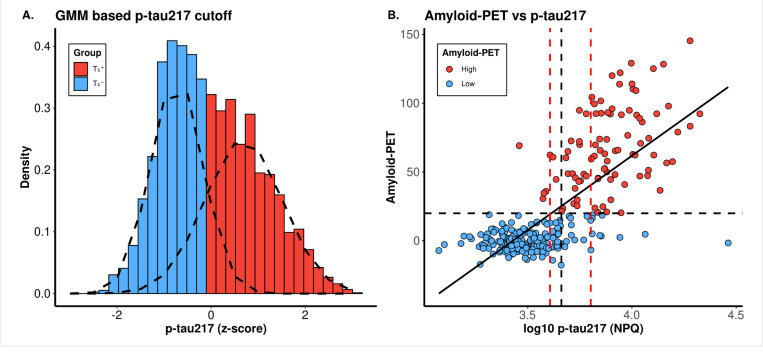
Identification of p-tau217 positivity cut off and its association with amyloid imaging. **(A)**The density plot shows the normal distribution of Z-scores from GMM using p-tau217 data. The x-axis represents Z-scores, with the red part for p-tau-positive samples and the blue part for p-tau-negative samples. The intersection of the colors marks the cutoff value. **(B)** The scatterplot illustrates a positive correlation between p-tau217 and amyloid-PET. The vertical black dashed line indicates Youden’s Index single cutoff for plasma p-tau217, which optimally balances sensitivity and specificity in distinguishing high (>20) from low (<20) amyloid-PET levels. The intermediate range of plasma p-tau217 is shown with the lower and upper vertical red dashed lines, representing the values associated with 95% sensitivity (lower line) and 95% specificity (upper line) for differentiating high (>20) from low amyloid-PET levels (<20). Out of 289 samples, 55 (19.03%) fall within the plasma p-tau217 intermediate value range.

We used a data driven model to identify the cut-off for biomarker positivity, following previous studies^29,40–42^. Specifically, we used GMM, including data from all samples (*N* = 3,659) to identify the cut-off. Through this approach, we established the p-tau217 z-score cutoff at −0.06 (corresponding log_10_ p-tau217 NPQ level: 3.66; or linear *NPQ* = 4,582.42; Fig. 4A). Based on this cutoff, 1,913 samples were classified as biomarker-negative (T_1_^−^), while 1,746 were classified as biomarker-positive (T_1_^+^). The Youden Index^43^ was also tested to determine a single cutoff, which matched GMM-derived value when rounded to two decimal places.

A confusion matrix was constructed from samples with both p-tau217 levels and amyloid-PET information (*N* = 289) to assess the p-tau217 cutoff concordance with amyloid-PET positivity. The overall concordance rate between p-tau217 and amyloid-PET-based biomarker status was 90.31% (28 misclassified participants), in good agreement with the literature value of 90% (Fig. 4B, Supplementary Table 9)^43^. This analysis also yielded an AUC of 0.95 for plasma p-tau217 vs. amyloid-PET. (*PPV*:0.82 and *NPV*:0.95; Supplementary Table 10).

Previous research has suggested that a two-cutoff approach, which includes an intermediate class of samples, outperforms a single-cutoff approach.^43^ We tested this by using p-tau217 levels at 95% sensitivity and 95% specificity as the lower and upper cutoffs. Samples were categorized into low (*N* = 74 (25.61%), log_10_-p-tau217 < 3.61), intermediate (*N* = 55 (19.03%), log_10_-p-tau217 = 3.61−3.8), and high (*N* = 160 (55.36%); log_10-_p-tau217 > 3.8). We then checked concordance by only including the high and low classes. The concordance rate improved from 90.31% in the single cutoff classification to 93.59% in the double cutoff approach (Supplementary Table 9).

We also evaluated the correlation between plasma p-tau217 and Tau-PET, observing a moderate association between the two (*r* = 0.39, *p* = 2.36×10^−10^; Supplementary Fig. 5A-D). Applying the same two-threshold cutoff approach yielded a 93.01% concordance of p-tau217 with and Tau PET (Supplementary Fig. 5A; Supplementary Table 9) and an AUC of 0.93 when adjusted with age and sex (95% *CI*:0.89−0.98), which is lower to that for amyloid-PET (Supplementary Fig. 5B, 5C, Supplementary Table 10). Without adjusting for age and sex, the AUC for Tauopathy is 0.93, which is slightly lower than that for amyloid-PET (*AUC* = 0.95; Supplementary Fig. 5D).

Finally, we examined whether the correlation between p-tau217 and tau-PET results was influenced by all individuals or primarily by those who were amyloid or Tau-PET positive. The analysis revealed that the correlations were predominantly driven by biomarker-positive individuals (*r*_*Amyloid*_= 0.434, *r*_*Tau PE1*_= 0.508), rather than biomarker-negative individuals. (*r*_*Amyloid*_= 0.164, *r*_*Tau PET*_ = 0.027; Supplementary Fig. 6A-F)

### Identifying proteins associated with progression to symptomatic AD

**Figure 5:**
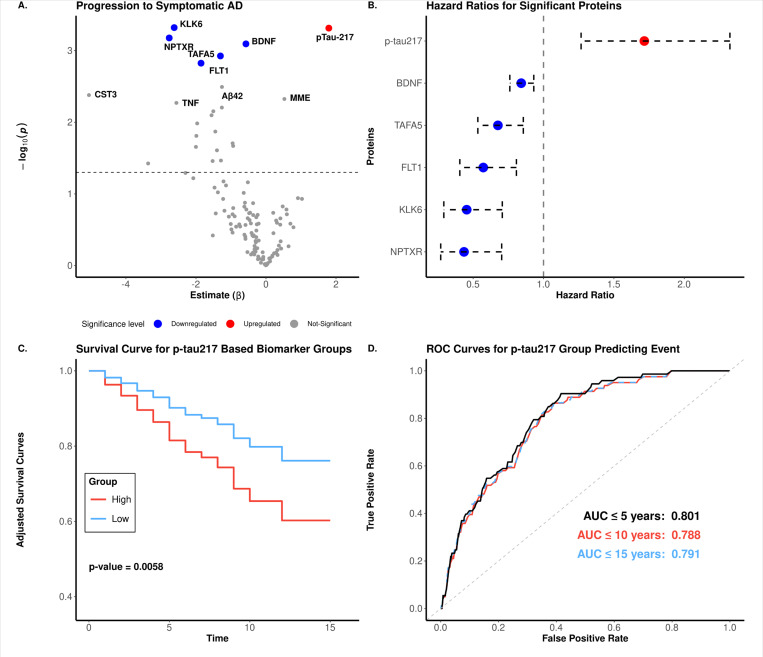
Proteins associated with progression to symptomatic AD. **(A)** Differential abundance analysis compares individuals initially classified as CO who either converted to AD or remained CO. In the volcano plot, colored dots indicate p-adjusted values < 0.05. **(B)** Whisker plot of hazard ratios and 95% confidence intervals for all significant proteins in Panel A. **(C)** Kaplan-Meier survival curves show the progression to AD following the initial blood draw. The biomarker-low group (blue line) and the biomarker-high group (red line) illustrate the proportion of participants who remain cognitively normal over the years of follow-up. The p-value highlights the significant difference in AD progression between individuals predicted to have AD and controls, based on the prediction model. **(D)** Receiver Operating Characteristic (ROC) curves assessing the predictive power of p-tau217 in classifying clinical Alzheimer’s disease (AD) over 5, 10, and 15 years.

Among the 1,579 CO participants at the time of plasma collection, 952 underwent follow-up clinical assessments in AD research. Of these, 84 progressed to symptomatic AD over a follow-up period of one to 23 years. We used these data to identify proteins associated with AD progression. Using a Cox proportional hazards model, adjusted for age at blood draw and sex, we identified six proteins that were nominally associated with disease progression (Fig. 5A). BDNF (hazard ratio *(HR)* = 0.84, *p* = 2.47×10^−2^), KLK6 (*HR* = 0.45, *p* = 2.47×10^−2^), NPTXR (*HR* = 0.44, *p* = 2.47×10^−2^), TAFA5 (HR = 0.68, *p* = 2.92×10^−2^), and FLT1 (HR = 0.57, *p* = 3.07×10^−2^, Fig. 5A–B, Supplementary Table 11) exhibited significant protective associations, indicating their potential roles in reducing the risk of developing symptomatic AD when accumulated.

In contrast, higher p-tau217 levels were associated with an increased risk of progression to symptomatic AD (*HR* = 1.71, *p* = 0.48×10^−4^, Fig. 5A–B). Over a 15-year period, subjects with higher p-tau217 levels were more likely to progress to AD compared to subjects with lower p-tau217, 80% of whom remained disease-free within the timeframe (*p* < 0.01, Fig. 5C) with an AUC of 0.801 when predicting subjects that progressed to AD within 5 years (Fig. 5D). For longer durations, the predictive power for those progressing to symptomatic AD at 10 and 15 years was 0.788 and 0.791, respectively (Fig. 5D). Together, these findings underscore the potential of p-tau217 in monitoring disease and predicting symptom onset in pre-symptomatic individuals.

### Pathway analyses highlight shared and disease-specific pathways

**Figure 6:**
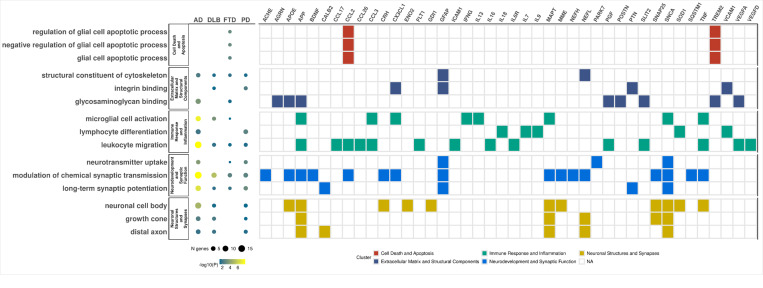
Disease-specific pathway analysis. **(Left)**The dot plot presents selected pathways across patients with Alzheimer’s disease (AD), dementia with Lewy bodies (DLB), frontotemporal dementia (FTD), and Parkinson’s disease (PD). Clustering of these selected pathways is indicated in the notation on the left side. The size of each dot represents the number of identified genes, while the color reflects the significance adjusted for false discovery rate (FDR). **(Right)** The tile plot highlights differentially expressed genes within each pathway. Color coding corresponds to the specific cluster to which each pathway belongs.

We performed pathway enrichment analysis using the proteins associated with the four studied disease. Overall, pathway analysis indicated that these proteins are primarily associated with four central mechanisms in all neurodegenerative diseases:1) neurodevelopment and synaptic function, 2) immune response and inflammation, 3) neuronal structures and synaptic integrity, and 4) extracellular matrix organization and structural components (Fig. 6; Supplementary Fig. 7; Supplementary Table 12).

The neurodevelopment and synaptic function included several sub pathways like long-term synaptic potentiation (*p* < 1.90×10^−2^), modulation of chemical synaptic transmission (*p* < 2.73×10^−3^), and neurotransmitter uptake (*p* < 4.55×10^−2^; Supplementary Table 12). The proteins associated with these pathways included GFAP, APP, SNCA, SNAP25, SQSTM1, MME, APOE, and CRH, among others (Supplementary Table 12).

Immune response and inflammation included the sub-pathways leukocyte migration (*p* < 3.89×10^−2^), lymphocyte differentiation (*p* < 1.06×10^−2^), and microglial cell activation (*p* < 4.67×10^−2^; Supplementary Table 12). Key proteins identified in these pathways were cytokines (ILs, TNF,15, IFNG, CCLs), vascular factors (VEGFD, VEGFA) and others (SLIT2, ICAM1 APP, TREM2, PTN, SOD1). In addition, distal axon (*p* < 2.90×10^−2^), growth cone (*p* < 4.32×10^−2^), and neuronal cell body (*p* < 4.31×10^−2^; Supplementary Table 12) were some of the major pathways that comprised the neuronal structures and synaptic integrity super pathway. MAPT, NEFL, APP, SNCA, and SNAP25 were the proteins that drove the association for these pathways. Finally, we identified key pathways associated with extracellular matrix organization and structural components including integrin binding (*p* < 4.17×10^−2^), structural constituent of cytoskeleton (*p* < 3.12×10^−2^), and glycosaminoglycan binding (*p* < 3.54×10^−2^: Supplementary Table 12). The pathways associated with the structural constituents of the cytoskeleton prominently include proteins such as GFAP, NEFL, AGRN, and NEFH, frequently occurring in combinations with GFAP and NEFL.

In addition to these shared pathways, disease-specific pathways were also identified. Pathways associated with receptor and ligand binding/signaling, including vascular endothelial growth factor receptor binding (*p* = 3.21x10^−4^), were distinctive to AD and predominantly driven by VEGFD and VEGFA (Supplementary Fig. 7). Cell death and apoptosis pathways were unique to FTD and driven by CCL2 and TREM2. These included the glial cell apoptotic process (*p* = 2.39×10^−3^), negative regulation of glial cell apoptotic process (*p* = 1.37×10^−3^), and regulation of glial cell apoptotic process (*p* = 1.37×10^−3^, Supplementary Fig. 7). Pathways related to enzymatic activity and metal ion binding, such as carbon-oxygen lyase activity (*p* = 2.17×10^−2^) and hydro-lyase activity (*p* = 1.53×10^−2^), were unique to PD, primarily driven by ENO2 and PARK7 (Supplementary Fig. 7).

In summary, we corroborated that major pathways previously implicated in neurodegeneration were replicated in our study and were driven by the most significant proteins from the abundance analysis.

### Technical properties and orthogonal validation of NULISA assays

First, we assessed the reproducibility of NULISA measures by running the same set of samples in two separate runs on different dates. (*N* = 86, Supplementary Table 13). The average correlation observed was 0.75 (Supplementary Fig. 8A) with 69.92% (86 out of 123) analytes showing a correlation higher than 0.7 (Supplementary Table 14). Neurofilament Heavy Chain (NEFH) showed the highest reproducibility with correlation between runs being 0.98 (*p* = 5.28´10^−56^). Other proteins that demonstrated high reproducibility included NRGN (*r* = 0.98, *p* = 4.15´10^−56^), FGF2 (*r* = 0.97, *p* = 7.73´10^−54^), GDNF (*r* = 0.97, *p* = 1.47´10^−51^), MME (*r* = 0.97, *p* = 1.93´10^−52^) and ANXA5 (*r* = 0.97, *p* = 7.45´10^−52^) among others (Supplementary Table 14). The AD/ADRD-related biomarkers - p-tau217, p-tau181, p-tau231, TREM2, GFAP, NRGN, and NEFL - all exhibited correlations greater than 0.87 (Supplementary Table 14, 15).

We found that proteins with high correlation across runs also exhibited higher correlation in different matrices (Supplementary Fig. 8B). Further analysis showed that proteins with higher correlations across runs had higher IQRs, while those with lower correlations had lower IQRs (Supplementary Fig. 8C; Supplementary Table 16, 17). These findings suggest that proteins demonstrating consistent reproducibility also exhibit high biological variability.

EDTA is commonly used for hematological exams, with many blood samples anticoagulated with it. Sodium citrate is preferred for coagulation studies due to its reversible effect with Ca2+ addition.^44^ In this study, we measured 43 samples using the NULISA CNS panel with two sample stabilization additives, EDTA and sodium citrate (*N* = 43; Supplementary Table 13). We found an average correlation of 0.67 between the runs, with 53.66% (66 out of 123) analytes with correlation of 0.70 or higher (Supplementary Table 14, Supplementary Fig. 8D). The highest correlation was observed for NEFH (*r* = 0.99, *p* = 5.21´10^−37^) followed by GDNF (*r* = 0.99, *p* = 1.28´10^−31^, Supplementary Table 14). The AD/ADRD biomarkers p-tau217, p-tau181, p-tau231, TREM2, GFAP, and NEFL all had correlations >0.84. Oligo-SNCA showed a negative correlation (*r* = −0.05), but it was not statistically significant. (Supplementary Table 14).

To further perform technical validation of the NULISA assay, we leveraged proteomic data for samples measured using immunoassay techniques (*N* = 1,414) and SomaScan (*N* = 3,622; Supplementary Table 18). Immunoassay measurement was available for p-tau181, Aβ40, Aβ42, GFAP, NEFL. We observed high correlation for GFAP (*r* = 0.83, *p* = 6.6 ´10^−139^), NEFL (*r* = 0.81, *p* = 1.6´10^−126^), and p-tau181 (*r* = 0.71, *p* = 2.5´10^−88^) between NULISA and immunoassay measurements (Supplementary Fig. 9) as previously reported.^15^ Moderate correlation for Aβ40 (*r* = 0.63, *p* = 1.1´10^−159^) and Aβ42 (*r* = 0.50, *p* = 2.1´10^−91^) was observed.

Of the 123 NULISA CNS proteins, 112 are also included in the SomaLogic 7K panel (148 total analytes pairs, as Somalogic has more than one assay for the same proteins). Overall, 125 analytes showed a significant correlation (*p* < 0.05; Supplementary Table 19), and the mean correlation between the platforms was 0.29 (Supplementary Table 19, Supplementary Fig. 10). Eight proteins pairs showed a correlation higher than 0.8, and 25 proteins pairs higher than 0.7. The highest correlation was observed for CHIT1 (*r* = 0.92, *p* <10^−300^) followed by TREM2 (*r* = 0.87, *p* < 10^−300^; for all aptamers; Supplementary Fig. 10). Other known markers of neurodegeneration NRGN and CHI3L1 showed correlations of 0.77 (*p* < 10^−300^) and 0.62 (*p* < 10^−300^) respectively (Supplementary Table 19, Supplementary Fig. 10).

Overall, we observed that the majority of NULISA assays produced reliable results across runs, with assay-dependent high correlation identified between NULISA and other proteomic platforms.

### NULISAseq classification of *APOE* genetic status

The *APOE e4* allele is the most significant genetic risk factor for AD; a single e4 allele increases disease risk by two to four fold^45^. An early determination of *APOE e4* carrier status can help AD risk management through targeted therapies and lifestyle changes. NULISAseq CNS Panel includes an assay targeting the APOE e4 proteoform, informing on whether an individual expresses the e4 allele. Of the 3,668 samples measured using the NULISAseq platform, *APOE* genotype information was available in 3,562 samples through direct SNP genotyping (Taqman). The presence or absence of variants rs423958 and rs7412 was used to infer *APOE* status. Individuals with genotype of ε2/ε2, ε2/ε3, ε3/ε3 alleles were labelled as *APOE4*− and those who had ε2/ε4, ε3/ε4 or ε4/ε4 genotype were considered *APOE4+.*

We observed a concordance rate of 99.27% between NULISAseq and genotyped-based *APOE* status assignment (Supplementary Table 20). Among the 1,457 samples assigned as *APOE4*+ through genotyping, NULISAseq confirmed that 1,443 expressed the e4 isoform, while remaining 14 samples were classified as *APOE4-.* Of the 213 samples genotyped as homozygous e4, NULISAseq labeled all but one as *APOE4+,* achieving 99.44% concordance within the group (Supplementary Table 21). Participants who were homozygous s3 carriers accounted for 13 of the 14 total mismatches. Similarly, NULISAseq correctly classified 99.42% (2093 of 2105) of *APOE4*−individuals, as determined through genotyping, as lacking the ε4 isoform. Of the 12 samples incorrectly labeled as *APOE4+,* 11 were ε2 homozygous, and one was ε2/ε3 heterozygous. Despite correctly labeling 99.22% (3,197 of 3,222) of ε3, most of the discordance was driven by samples carrying at least one ε3 allele (14 genetic APOE4-labelled as APOE4+ and 11 genetic APOE4+ labelled as APOE4-). Overall, these results demonstrated a robust concordance of classifying *APOE s4* status between genotyping and NULISAseq.

## Discussion

In recent decades, significant progress has been made in discovering novel biomarkers of AD and ADRDs.^7,46,47^ Blood-based biomarkers are attractive due to the simplicity of sample collection and the potential for widespread adoption.^48–50^ The advent of sensitive, multiplexed, and indirect measurement proteomic platforms has further enhanced the potential utility of these fluid biomarkers. Larger studies interrogating the accuracy, sensitivity, and specificity of these assays are necessary to validate and instill confidence in their readouts. In this context, our study has evaluated the recently developed multiplex NULISA technology.^15^ We analyzed 123 CNS-specific proteins in 3,668 plasma samples (3,002 unique participants), marking a threefold increase in sample size compared to previous studies.^50–52^ The cohort included samples from participants with ADRDs (PD, DLB, FTD, and AD) and cognitively normal controls. Participants were well-characterized, with AD-related phenotypic measurements such as amyloid-PET, Tau PET, Aβ42/Aβ40 ratio, and CDR. Finally, we compared the protein measurements from this platform with legacy measures such as single-plex immunoassay and an orthogonal multiplex platform, i.e., SomaScan.^34^

Plasma p-tau217, GFAP, NEFL and PARK7 were identified as the most significant associated proteins in AD, DLB, FTD, and PD, respectively, compared to cognitively normal individuals.(Fig.1). As p-tau217 was the most significant AD-associated protein, we analyzed its biomarker performance in the context of AD-related phenotypes such as amyloid-PET, Tau-PET, Av42/40 ratio and CDR. The direction of the association was consistent with known AD pathophysiology, with an increased plasma p-tau217 associated with higher amyloid-PET, Tau PET, and CDR values and a lower Ab42/Ab40 ratio.^53–56^ Plasma p-tau217 showed very high predictive power for clinical AD status (*AUC* of 0.81) and amyloid-PET positivity (*AUC* of 0.95), consistent with previous reports that p-tau217 describes amyloid pathology.^57^ We also analyzed its predictive power for amyloid and Tau-PET and observed similar AUC for both traces, with a slightly higher AUC for amyloid (*AUC* = 0.95) than Tau-PET (0.93; Supplementary Fig. 6). In general, our findings are in line with what has been reported in recent studies and highlight the utility of plasma p-tau217 in predicting AD pathology, particularly those characterized by amyloid changes.^58,59^

As the largest study using the NULISAseq CNS Disease Panel 120, we established the biomarker positivity cutoff for plasma p-tau217 through multiple data-driven methods. Single-threshold cutoff approaches, utilizing the Youden Index or GMM (linear NPQ > 4582.42; log10 p-tau217 NPQ > 3.66), showed high concordance (90.31%) with amyloid-PET, aligning with findings from a recent study.^43^ Applying a two-cutoff approach further enhanced p-tau217’s predictive performance and improved alignment with amyloid-PET classifications in overlapping samples. Given the high cost and specialized nature of PET imaging required to determine amyloid-PET values, plasma p-tau217 could be a cost-effective pre-screening tool for imaging analyses.

In our study, p-tau217 was also associated with an increased risk of progressing to AD. In fact, individuals with higher levels of p-tau217 were more likely to progress to AD within 15 years compared to those with lower levels. By contrast, five proteins (NPTXR, KLK6, BDNF, TAFA5 and FLT1) were associated with decreased risk of developing disease and overall were negatively associated in AD subjects compared to cognitive normal individual (Fig. 5, Supplementary Table 1). Previous studies have shown that NPTXR levels are reduced in the CSF and plasma of individuals with AD compared to cognitively normal individuals.^60,61^ NPTXR is a protein primarily found in neurons and plays a key role in synapse organization, and altered levels can also be detected in plasma, making it a potential biomarker for distinguishing AD from controls, as demonstrated in a recent study.^37^ KLK6 is involved in the regulation of neuronal function and the breakdown of amyloid-beta plaques in Alzheimer’s disease^37,40^. Our results closely align with a previous study in over 600 individuals with mixed dementia diagnoses and controls that reported plasma neurosin (Kallikrein 6, hk6) levels increase with age in healthy individuals but decrease in patients with AD.^62^ Similarly, a recent NULISA-based study found increased plasma KLK6 was linked with a slower rate of neurodegeneration in a preclinical AD cohort.^63^ Conversely, increased plasma KLK6 levels have also been reported in patients with more advanced stages of AD, which we did not investigate.^64^ Overall, our findings highlight proteins that may exhibit protective effects against AD.

We also observed a clear binomial distribution of p-tau217 levels in DLB, FTD and PD (Fig. 2), overlapping with that in controls and some AD cases. This binomial distribution is more accentuated in DLB, which is likely capturing the Aβ pathology also found in DLB cases.^65,66,67,68^. It is important to consider that individuals with FTD or PD may also exhibit amyloid positivity^69^, which is common at the population level in these diseases, even if AD is not the primary driver of their dementia. This amyloid pathology could help explain the observed high p-tau217 levels in these cases, further supporting the notion of pathological overlap.

In AD, besides p-tau217, there were 77 associated proteins. These included known AD and neurodegenerative biomarkers such as p-tau231, GFAP, Ab42, NEFL, NPTXR among others. GFAP, which is a marker of reactive astrogliosis, and NEFL, which is a marker of neuroaxonal damage, were the only proteins also associated with DLB and FTD.^70^ Both markers have been previously reported to have an exceptional performance in monitoring cognitive changes in DLB^70^. A previous study highlighted the importance of clinical history and visuospatial function assessment, rather than spontaneous extrapyramidal signs (EPS), in differentiating DLB from AD in early stages.^71^ While their approach focused on clinical features, our proteomic analysis revealed that AD and DLB share highly correlated profiles (*r* = 0.78) and similar dysregulated pathways, such as microglial activation (APP, CCL3, CX3CL1) and neuronal processes (APOE, MAPT, MME, SNAP25, SNCA). Importantly, proteins such as CRH, NRGN, CRP, and Oligo-SNCA exhibited opposite directions, offering potential for differential diagnosis. When extending these analyses and comparison across all the diseases, we found similar patterns, in which there is a large overlap and concordance across diseases, but some disease-specific proteins were evident. For example, PARK7, was PD-specific. PARK7 is a well know mediator of PD pathogenesis via mitochondrial dysfunction. Mutations in *PARK7* have been linked to familial forms of PD.^72^ Neprilysin (MME), a neutral endopeptidase involved in the immune response, shows increased levels specifically in FTD, as the effect size of this protein is highest in this disease compared to all others.

Pathway analysis identified processes associated with neurodevelopment, synaptic function and integrity, immune response, and extracellular matrix organization and structural components. These pathways were shared across all neurodegenerative diseases. Pathways unique to AD included vascular endothelial growth factor receptor binding whereas pathway associated with apoptotic processes of glial cells were uniquely enriched in FTD. The two proteins that were major drivers of this apoptotic pathway in FTD were CCL2 and TREM2. Increased serum CCL2 has been reported as potential aggravator of FTD pathology and *TREM2* mutation has been suggested with development of FTD like phenotype previously^73–75^, and low frequency variants in this gene have been reported to be associated with AD and FTD.^76–79^

To technically validate the performance of the NULISA platform, we compared analyte measurements orthogonal protein readouts, inter-day variations, and among plasma stabilization conditions, as well as *APOE4* genetic status. NULISA achieved an average correlation of 0.696 across the five plasma proteins p-tau181, Ab40, Ab42, GFAP, NEFL measured with immunoassays, and 0.29 across 112 commonly measured proteins (158 total comparison) with SomaScan. Known markers of AD and neurodegeneration like p-tau217, GFAP, CHIT1, NEFL, TREM2 and p-tau181 showed high correlation (>0.7) across all analysis. Comparison between runs within NULISA platforms also showed that GFAP, p-tau217, p-tau181, NEFH, and NRGN were highly correlated (>0.9). We observed that protein measure reproducibility was directly proportional to its IQR (Supplementary Fig. 8). Since this analysis involved a test-retest of the same samples, we interpret that highly variable proteins are likely harder to quantify due to low abundance or technical challenges associated with protein extraction and measurement processes, rather than reflecting biological differences at the subject level. Finally, NULISAseq correctly predicted the APOE4 genetic status of 99.27% of individuals for whom allele data were available, whereby carriers of at least one ε3 allele accounted for most of the missed classifications. These findings suggest that the multiplex assays in the NULISAseq CNS Disease Panel 120 are accurate, consistent, and sensitive to biological variability, making them ideal for blood collection-based disease monitoring and research settings.

Despite being the largest study to date that comprehensively evaluates the biological and technical aspect of the NULISA platform, this study has some limitations. First, while the study included four neurodegenerative diseases, the sample sizes for FTD, DLB, and PD were modest. Additionally, the highly heterogeneous nature of FTD may have impacted the uniformity of some signals, further emphasizing the need for larger sample sizes in future studies. Second, the current study utilizes racially homogenous samples with majority being non-Hispanic white. Follow-up studies with ethnic diversity are needed not only to assess plasma proteomic composition differences, but also to validate our findings. Third, since we utilized cross-sectional samples for our analysis, long term changes, trajectories, and stability of the biomarkers will need to be studied in longitudinal studies. Finally, our comparison of NULISA with other established proteomic platforms was constrained to the analytes measured using immunoassays or SomaLogic. Including additional neurodegeneration-related analytes will enhance the comprehensive evaluation of the platform’s accuracy.

Overall, our study highlights disease-specific and shared circulating signatures among neurodegenerative dementias. Plasma p-tau217 shows strong potential to predict amyloidosis, with a robust correlation to amyloid-PET levels, supporting its use as a population-level screening tool. These findings further cement the utility of multiplexed NULISA targeted protein quantification, particularly p-tau217 in neurodegenerative disease research and clinical applications.

## Supplementary Material

Supplementary material is available at *Brain* online

This is a list of supplementary files associated with this preprint. Click to download.

F04SupplementaryTablesManuscriptNULISAPlasmaADRC.xlsx

F03SupplementaryFiguresNULISAPlasmaADRC.docx

## Figures and Tables

**Table 1:** Demographic Information of Participants in the Disease Analysis

Group	*N*	Mean Age at Draw (SD)	Mean Age at Onset (SD)	Male (%)	*APOE4*+ (%)
**AD**	1,092	77.68 (± 8.37)	73.20 (± 8.84)	43.68	58.33
**DLB**	28	75.46 (± 11.57)	70.71 (± 11.64)	64.29	42.86
**PD**	9	77.11 (± 6.79)	72.67 (± 9.64)	88.89	33.33
**FTD**	39	67.28 (± 9.45)	63.10 (± 9.88)	56.41	46.15
**CO**	1,579	72.88 (± 10.58)	-	40.22	30.72

Table 1 summarizes the demographic characteristics of participants in the disease analysis, including the number of participants (N), mean age at the time of sample collection (Age at plasma Draw), mean age at disease onset (Age at Onset), percentage of males in the group (Male %), and percentage of participants carrying at least one APOE4 allele (APOE4+ %). Groups include individuals diagnosed with Alzheimer Disease (AD), Dementia with Lewy Bodies (DLB), Parkinson Disease (PD), Frontotemporal Dementia (FTD), and cognitively unimpaired controls (CO).

**Table 2:** Demographic Information of Participants in the Phenotype Analysis

Phenotype	*N*	Mean Age at Draw (SD)	Mean Age at Onset (SD)	Male (%)	*APOE4*+ (%)
**Amyloid-PET**	280	69.01(±8.35)	71.96(±9.02)	47.14	41.79
**CSF Aβ42/Aβ40**	504	68.23(±8.77)	70.20(±8.26)	44.05	41.47
**Tau PET**	264	68.00(±8.53)	67.40(±8.61)	45.45	41.67
**CDR**	2,671	74.84(±10.02)	74.20(±9.56)	41.63	42.01

Table 2 presents the demographic characteristics of participants included in the phenotype analysis. The table includes the number of participants (N), mean age at sample collection (Age at Draw), mean age at disease onset (Age at Onset), percentage of males (Male %), and percentage of participants with at least one APOE4 allele (APOE4+ %). Phenotypes analyzed include amyloid-PET values from amyloid PET imaging, CSF Aβ42/Aβ40 ratio, Tau PET imaging results, and Clinical Dementia Rating (CDR).

## Abbreviation

Aβ: β-amyloid
AD: Alzheimer disease
ADRD: Alzheimer’s Disease and Related Dementias
AUC: Area Under the ROC curve
CDR: Clinical Dementia Rating
DLB: Dementia with Lewy Bodies
FDR: False Discovery Rate
FTD: Frontotemporal Dementia
IQR: Interquartile range
NPQ: NULISA Protein Quantification
NPV: Negative predictive values
PD: Parkinson’s disease
PiB: Pittsburgh Compound-B
PPV: Positive predictive values
p-tau: Phosphorylated tau
ROC: Receiver operating characteristic
SV: Surrogate variable
